# Practical Observations and Suggestions in Medicine

**Published:** 1847-01

**Authors:** 


### Art. XIV.

*Practical Observations and Suggestions in Medicine.* By Marshall Hall, M.D., F.R.S., L. & E., &c. &c.—*London,* 1845-6. First series, 8vo, pp. 360; Second series, 8vo, pp. 360.

In our Number for April, 1845, we noticed a volume of 'Observations in Medicine,' by Dr. Hall. We *noticed* it merely, and out of kindness to the author, because we felt convinced that an impartial analytical and critical review of the volume, must necessarily detract from the reputation already acquired by Dr. Hall. The appearance of another and a similar volume recalls us to a juster sense of our duty,—reminding us that there is something due to the character of British medical literature and science, and that a tenderness for the faults of an able man ought not to render us disloyal to truth. We therefore now propose to review these 'Observations' more in detail.

In our previous notice, we said that a man with the reputation of Dr. Hall could not afford to bring out an inferior book, for his own sake: we now say that, for the sake of others, he must not be allowed to bring out one inferior book after another, unless a just estimate of them is laid before the profession at the same time. Example in high places is as potent for ill as for good; and the name of Dr. Hall may make that pass current which would be at once rejected under a lesser warranty. In criticising the volumes before us, we shall distinctly state the grounds of any opinion we may express regarding the author's merits; and, doing this, our readers will themselves be able to criticise the critic.

The author's objects in publishing these volumes would seem to be various; they have all, however, a reference either to the student or the practitioner. In the first place, Dr. Hall evidently desires to set himself up to both as an example and a teacher. This purpose discloses itself in the very beginning of the book. Among the "introductory remarks" of the first volume, in the section on "fertility in remedial resources," as a requisite in an able physician, we have the following:

> "If the following 'Observations and Suggestions' prove that I have not cultivated *this* subject entirely in vain, and lead other physicians into the same useful train of mental culture, I shall be amply rewarded for the pains which I have taken in embodying them in language and laying them before the profession.
>
> "I have published these 'Practical Observations and Suggestions' in an unpretending form: first, that they may appear in the modest manner becoming such a trifle; and, secondly, that they may be readily portable; for my ambition is, that they may become the companion of the student, and of the country practitioner, in some of his lonely drives, especially of those whose time is too much taken up by practice to admit of their reading larger and denser volumes.
>
> "I have also wished not to deter, by the form of this work, the general reader, who may be interested in medical matters generally, or in some particular medical subject." (Vol. i, p. 3.)

Another object appears to be, the promulgation of the author's therapeutical experience, or the communication of methods of treatment already found useful by him—such as the daily scarification of the gums in infancy, the alcoholic lotion in phthisis, &c.; or of hints or suggestions of treatment derived from analogies, or from physiological ratiocination, as the cold douche to the loins in sterility, the cure of vascular nævus by puncture, &c.

A third object of the author is the republication of essays formerly written on various subjects. Of this kind are the chapters on "intestinal irritation," "exhaustion from loss of blood," "the sinking state from various causes," "on hybernation," &c.

A fourth object is to make the work a sort of receptacle for the lucubrations of friends, and for their hints, observations, and suggestions. When we consider how freely open the numerous weekly and monthly journals are to the reception of communications of this kind, we think this altogether unnecessary, and at variance with the other objects of publication. The observations we allude to are certainly *not* "Observations by Dr. Marshall Hall."

Dr. Hall's volumes are also "intended to be suggestive of new observations, as well as the record of some observations of his own;" and, in giving these suggestions for new observations, the author indicates special objects of inquiry, propounds the principles of observation that should be adopted, and lays down the plan that should be followed. The subjects indicated for observation are detailed in vol. ii, p. vii.

The following extract exhibits the principles propounded by Dr. Hall as the guide to students and practitioners in conducting the suggested observations:

"I am persuaded that physiology, or a knowledge of the healthy actions, is the only foundation for practical medicine,—and the only remedy against the hydra, quackery, now so prevalent, both in and out of the profession. This physiology should be at once experimental and clinical. The biblio-physiology of the day can issue in no good whatever. The medical mind wants discipline; we should study the works of Harvey and of M. Louis; experiment, and observation, and philosophy should go hand in hand....... When every member of our profession has a sound knowledge of physiology, derived from his own cautious observation, and not, at second and third hand, from books, medicine will take its just position, and quackery its departure. But whilst a medical person can be found to boast that he, forsooth, is a 'mere practical man,' quackery will continue. One kind of mere empiricism is as good as another." (Vol. ii, pp. v, vi.)

These principles of clinical observation we cannot object to, as they are almost the same as some which were advocated in the correspondence elicited by an article published in this Journal last January, and particularly in one of the letters addressed to the editor in the Twenty-first Volume. We are happy to find that these views, although omitted in the first volume, find so prominent a place in the second; and we are the more gratified, because we can bring Dr. Hall's "purely useful and practical" observations to the test of his own avowed principles without any demur on his part. It is rather pleasing, too, to find our author laying down a "plan of observation of diseases of the nervous system," prefaced by this declaration: "If we wish to pursue the subject of *clinical* observation in general, we have fortunately a *perfect* model and example in the writings of M. Louis."

The value of the new therapeutical experience recorded by Dr. Hall varies extremely. As an example, we will take the first practical point in the first volume, and we think it will not be difficult to show, that the observations recorded in that chapter have not been made under the guidance of physiology, but that they are in absolute contrast to the "*perfect* model and example of M. Louis," whilst the whole chapter displays, as we shall subsequently point out, a spirit and aim very remotely related indeed to the lofty aspirations of the author. The chapter is headed, "On the use of the alcoholic lotion in phthisis pulmonalis."

"So many persons affected by incipient phthisis, marked by dulness of sound on percussion, and no doubtful pectoriloquy under the clavicle, hæmoptysis, and disposition to chills, heats, and early morning perspirations, &c. have been benefited and restored to apparent health by the remedy, or remedies, which I am about to mention, that I cannot but think they possess great efficacy. The first and the principal of these remedies is an alcoholic lotion constantly applied by means of six folds of linen over and across the upper lobes of the lungs. One part of pure alcohol is mixed with three parts of water. It is applied tepid at first, afterwards of the temperature of the atmosphere. It is applied, in *small* quantity at a time, every *five* minutes, so that the application may always consist of alcohol and water." (Vol. i, p. 25.)

On the next page we find the alcoholic lotion gets all the credit; but still Dr. Hall's practice is successful.

"It is by no means my wish to laud this remedy beyond its just value; but I have no hesitation in asserting that it possesses a power in checking the progress of the deposition and softening of tubercle in the lungs, beyond any other which I have ever tried. And the number of patients who have recovered from incipient phthisis under its use, and who, after many years, are still living, and in apparent health, induces me to express myself in strong terms in regard to its extreme value." (Vol. i, p. 26.)

Then follows the list of cases, much in the style of those which usually are found to clench quack advertisements: for example—

"One patient, who consulted me fifteen years ago, had dulness on percussion and pectoriloquy, and every other sign of incipient phthisis. He applied, and long wore, the alcoholic lotion, called it his 'breast-plate,' and is now a professor of ——— College.

"A lady, about 30 years of age, became affected with hæmoptysis, and displayed the physical signs and the usual symptoms of phthisis. She was enjoined the alcoholic lotion. It is fourteen years since it was first applied, and it is continued, or renewed, if ever suspended, to this day.

"I saw a young lady two years ago, one of a most consumptive family, affected with hæmoptysis, and with every threatening sign and symptom of incipient phthisis. I prescribed the alcoholic lotion, and the cough and hæmoptysis were removed, and every fear dispelled. It had already been proposed that this young lady should take a voyage to Madeira. She did so, continuing the lotion, and returned in apparent good health." (Vol. i, p 27.)

Now, if these general statements mean anything, they mean that certain individuals labouring under phthisis pulmonalis were *cured* by the use of this alcoholic lotion. They mean this, and nothing less. But an indistinct idea seems to have passed through the author's mind, that his *professional* reader might bring some common sense to bear on the subject, and demur to the facts. We therefore have him stating, "I do not imagine that the alcoholic lotion does more than *check* the morbid processes." Nay, he goes further; he even doubts whether it even effects this:

"In what the morbid processes of the deposition and the softening of tubercle consist, I believe we do not know; but if these processes be really checked by the application of the alcoholic lotion, we have a *practical* fact which must excite the deepest interest. Some degree of this influence, in incipient cases, is, I believe, exerted by this remedy." (Vol. i, p. 28.)

What, then, is the therapeutical experience in this chapter? Firstly, Dr. Hall has no hesitation in *asserting*, that the alcoholic lotion possesses the power of checking the progress of the deposition and softening of tubercle; nay, "a number," and "so many," have been "restored" by it from what he calls "incipient phthisis," but which we must call confirmed phthisis, seeing there was "dulness of sound and no doubtful pectoriloquy under the clavicle"—that he cannot but "express himself in strong terms in regard to its extreme value;" and then, on the next page, all this is softened down into "*if* these processes be really checked by the alcoholic lotion," and "*some* degree of this influence is, I *believe*, exerted," &c. *I believe!—some!—if!*—are these the terms to use regarding a remedy for an incurable disorder after "*I have no hesitation in asserting?*"

We have scanned this chapter carefully, hoping to discover the slightest hint of the numerical method, or the smallest modicum of physiological science. We trusted that there might be at least one line which would indicate, however so obscurely, the physiological principles which had led Dr. Hall to the use of the remedy, and which would elucidate its *modus operandi.* But the result of our most careful analysis is, in the language of the chemist, *not a trace.* As for the "perfect model and example of Louis," we need only observe, that they are just as utterly wanting as the principles of physiology. Even the language is singularly loose and indefinite. For example, the folds of linen are ordered to be applied "over and across the upper *lobes of the lungs:* a mode of wording, moreover, as if it were meant to indicate the rationale of the practice. To say nothing of the next-to-impossible feat of applying the remedy "every five minutes,"—yet one lady seems to have done this for fourteen years!—not a hint is given as to how the influence of the alcohol reaches the morbid tissues, and when there, how it effects the beneficial change. There is not, we repeat, the slightest attempt at a physiological or numerical consideration of the nature of tubercle, or of the means by which the deposit may be prevented or removed. Yet, surely, if the "*perfect* model" and example afforded by Louis be ever worthy imitation, it is in investigating the nature and cure of phthisis pulmonalis.

There is a chapter "on the treatment of lateral curvature of the spine," and here we made sure of physiological observation. The pathology of spinal muscular action is so manifestly an important branch of the "true spinal" doctrines, that we were warranted in fully expecting from Dr. Hall an application of those doctrines to the pathology and therapeutics of lateral curvature. What Dr. Hall has really done shall be here stated:

"The plans for the treatment of curvature of the spine which I propose, have three objects in view:—The first is the restoration of the natural form. The second, the renutrition of the weak and emaciated muscles. The third, the restoration of the health and vigour of the general system. I propose to accomplish my first object by stays applied to the *curvatured* form, so constructed as to give the most perfect support without inducing the least injurious pressure, either on parts too protuberant, or on others, as the axilla or the ilium, taken as points of support.

"Let the patient be artificially made to assume the straight or perfect form by means of posture, stretching, and pressure; under these circumstances, carefully preserved, let a cast of the bust be taken in plaster of Paris; from this cast let a mould in wax be taken; lastly, on this mould let stays of steel be accurately fitted (or let copper be deposited by means of the electrotype and sawn in two vertically). This is to be covered and lined with soft leather and slight wadding, and fixed and worn in the ordinary manner, drawn to the proper degree of tightness.

"It is obvious that, these stays being put on and fastened in the recumbent posture, the bust *must* retain the perfect form, although the patient may now resume the erect position; that no partial or injurious pressure will be made on any part; and that there is neither any constrained position, nor any obstacle to the due use of the muscles." (Vol. i, pp. 55-6.)

All this is apparently theoretical: if Dr. Hall can produce the cases in which he has so applied the stays, and successfully, we trust that he will do so. A reference to the general practitioners in attendance—whom he is always so careful to name—will suffice. The next step in the treatment is thus described:

"In order to accomplish the restoration of nutrition in these atrophied muscles, something more must be done; and this remark leads me to a second proposition for the treatment of these distressing cases. It is that of inducing, by means of rubbing, what I will designate *counter-muscular effort.*

"If, whilst the patient is sitting perfectly erect and unsupported, we press on any given point along the spine with the finger, every [?] muscle situated *below* the point of pressure is necessarily [?] called into a state of action, which (action and reaction being equal, but in contrary directions) is, in degree, commensurate with that of the pressure, unless, indeed, the patient *yields* to the pressure. In this manner, any one of the numerous muscles situated on either side of the spinal column may be called into action *at will,* [?] and this by means of the very friction which was formerly used in a form that may be termed *inert,* and in a degree proportionate to the *pressure* with which it is applied.

"The patient being placed unsupported in the erect position, the hand or hands are to be passed along the muscles of the spine, pressing, at first very moderately, then more and more firmly, whilst they are carried upwards and downwards alternately, in the ordinary manner of rubbers. At every successive instant a fresh set of muscles is called into action more particularly, whilst the whole system of the spinal muscles is made to contract together, or in their turn." (Vol. i, pp. 58-9.)

Dr. Hall would have his readers infer a therapeutical novelty here; it is, however, nothing more than the old method of shampooing, kneading, &c. combined with gentle friction: friction alone is rarely, if ever, recommended by professional men. The physiological novelty is in the explanation of the *modus operandi,* which is, in truth, a most improbable hypothesis, namely, that on pressure being made on any given point along the spine, action takes place in every muscle situate *below* the point of pressure in *a degree proportionate to the amount* of pressure. In the treatment of curvature of the spine it is necessary to anything like success, to ascertain the exact muscles implicated in the distortion; then the nature of the morbid muscular action; and then the causes. Distortions of the bones, joints, or muscles usually, if not always, take place from unequal muscular action, consequent upon either disease of the bones to which the muscles are attached, or upon paralysis or spasm of the muscles themselves. The antagonism between the opposing muscles, either of the two halves of the body or of the two sides of a limb, is destroyed. We have obvious examples of this in distortion of the face, in wry-neck, in club-foot. Now, no one ought to know better than Dr. Hall, that this interruption of equilibrium of muscular action may arise from two opposite conditions of the muscles implicated. Firstly, there may be spasmodic action, as in spasmodic tic douloureux, and the distorting traction is in the direction of traction of the affected muscle. Secondly, there may be debility or paralysis, and then the healthy unparalysed muscles drag their movable attachment in the direction of their traction, as in facial paralysis. But there may be a third condition, namely, that in which there is spasm of the one set of muscles, and paralysis of the antagonizing set. The diagnosis in these several examples must often be difficult, and there could be no greater service rendered to practical medicine than an easy method of diagnosis, founded on just physiological data. When, for example, in strabismus, is there spasm, and when paralysis? or when the one condition or the other in facial distortion, or in spinal curvature? If we trace the different forms of spinal distortion downwards, we shall find the first form to be that in which the recti antici and longus colli seem to drag the occiput and atlas forwards, causing a corresponding depression in the nuchal region, and a projection in the lower cervical. In torticollis, sometimes one half of the trapezius is implicated, and always the sterno-cleido-mastoideus. Coming to the thoracic region—the more ordinary seat of scoliosis or lateral curvature—it is to be supposed that as the sterno-mastoid and trapezius were the distorting agents above, some of the same class—the respiratory class—of muscles will be the agents in the distortion below, either as healthy, unantagonized muscles, or as being spasmodically affected. It is maintained by some pathologists, that in scoliosis, or lateral curvature, the external respiratory muscles are those most usually affected, namely, the trapezius, sterno-cleido-mastoideus, levator anguli scapulæ, rhomboidei, scaleni, and the serratus magnus. Now if these be the muscles affected in ordinary cases of scoliosis, the pressure on the spinal muscles recommended by Dr. Hall, can be of no possible benefit, even if it be fully granted that shampooing, so performed, excites the sub-lying muscles proportionately to the pressure. But on what grounds does Dr. Hall assert that such a result takes place in the atrophied muscles of the spine? No doubt slight muscular contractions may and do occur; and we will give Dr. Hall the full benefit of this admission, and of his views as to the class of muscles affected, simply for the sake of asking him *which side,* in scoliosis, should be pressed and rubbed? for it is evident that if *both* sides be equally excited, the healthy, strong muscles will acquire increased bulk, at least, *pari passu* with the paralysed or enfeebled, and there will still be that unequal muscular action which originally caused the deviation of the spinal column. We will say nothing of the distortion which takes place from unequal action of the two latissimi dorsi, and of the muscular masses on each side the lumbar vertebræ, and attached to them and to the sacrum.

Dr. Hall finally asserts, respecting his plan of treatment—

"By the stays already described, the morbid extension is removed from the enfeebled, atrophied, and stretched muscles. By the friction and the counter-effort of these muscles they are nourished and strengthened. By both these means they are rendered efficient in their office of supporters and movers of the spinal column and the head." (Vol. i, p. 59.)

Now, we venture to affirm—1. That the stays, as recommended by him, will and must press injuriously, because, being inflexible, they cannot be adapted to the flexible spine; being inelastic, they cannot be adapted to the varying tension of the muscles. 2. That in the majority of cases of lateral curvature, the deviation is caused by the morbid action of varying sets of spinal muscles: in some cases, the external respiratory, or thoracic; in others, those of the dorso-lumbar region; and, consequently, that the hap-hazard shampooing recommended by Dr. Hall is, theoretically, of doubtful value. 3. That as the method has been repeatedly tried with so little success, we shall be not a little surprised if Dr. Hall can prove that the results above described by him as consequent on his plan of treatment ever occurred, even in a single case.

We will take one other of these practical papers, and we select the chapter on aphoria, or sterility; to which we will confess that we were attracted because it concludes with one of those random and ridiculous claims to discovery and novelty in which Dr. Hall indulges beyond any other man.

The pathology of aphoria is a subject on which a sound physiology only can throw light; and Dr. Hall accordingly observes, that it is by a knowledge of the physiology of conception, and of the causes that most probably influence that function, that we can be led to a suitable method of cure.

We believe that the doctrine of *ovarian* influence and sympathy, as opposed to the old doctrine of *uterine*, is now well established. It is certain, moreover, that the relations of the ovaria to the uterus, and to distant organs in connexion with the reproductive system, as the mammæ, are much better understood, and much more strictly defined. It has been amply proved that the ovaria are the essential organs of repoduction, and the point of departure of the greater proportion of those sympathies which have been usually attributed to the uterus. Thus it has been found, that extirpation, or non-development, or atrophy of the ovaria, is followed by a corresponding change in the characteristics of the sex, inasmuch as the uterus becomes atrophied, menstruation ceases, the mammæ waste, the subcutaneous fat disappears, the skin becomes harsher, the voice hoarser, and in some examples of a peculiar condition of the ovaria, even the characteristics of the male are developed. But the uterus may be extirpated or non-developed, and none of these changes occur, provided the ovaria are unchanged in structure or function. Even a secretion analogous to the menstrual fluid will occur at the regular periods, apparently from the vagina. The phenomena of menstruation specially have so close a causal relation with the ovaria, that it is probable no functional change in that secretion takes place without the agency, direct or *indirect,* of the ovaria. Structural changes, as ulceration, fibrous tumour, or scirrhus, have of course their own proper influence.

Now, is it not most certainly to be expected that a physiological physician, with pretensions like those of Dr. Hall, should at least convince his readers that he was fully acquainted with the whole physiology of conception, in discussing the pathology and treatment of barrenness? But what is the fact? This: that the whole of his chapter on this subject is written as physicians wrote a century ago—or rather is not so well written.

"Sterility, doubtless, frequently depends on organic defect; but the fact that a first child has been born after many years of marriage is a sufficient proof that, in other cases, the defect has arisen from causes of a functional and less permanent nature. Of these, a too excited condition, and the opposite state of inertia in the immediate uterine system, appear to me to be the most frequent. The former case is illustrated by what is observed in dysmenorrhœa; the latter, by a condition of the uterus, frequently attended by leucorrhœa. They may be designated aphoria tonica, and aphoria atonica, respectively." (Vol. i, p. 150.)

The treatment is deduced from empirical conditions. The families of the laborious poor are numerous and tend to augment the population, whilst those of the indolent and luxurious rich continually tend to become extinct. The conclusion, as Dr. Hall observes, is obvious; "as a remedy for aphoria, much exercise should be taken, even to fatigue, whilst the diet should be moderate, not to say, spare."

But surely such mere empiricism as this, or such as the recommendation of extreme moderation in intercourse, required no preliminary flourish about the "physiology of conception." The treatment is deduced from experience alone, and would be deduced just as certainly if Dr. Hall were in profound ignorance of the very existence of the ovaria and uterus. The poor multiply; if the rich wish to multiply, let them live like the poor. If they take humble fare and work hard, they will then digest well, sleep well, and have neither time nor inclination to waste their hours in enervating dalliance. But the advice is just as applicable to the rich *man* as the rich woman; for impotence, we suspect, is almost as common as sterility.

"But I proceed to another and most interesting view of this subject. There is an extraordinary reciprocal sympathy between the mammæ and the uterus, which has always presented an object of deep interest to the physiologist. At every catamenial period, the mammæ sympathise with the uterus, become tumid, and tend to assume their office of organs for the secretion of milk. But the sympathy is not only seen in the affection of the mammæ, by the condition of the uterus, but, though somewhat less definitively, in the inverse order, and the state of the mammæ influences that of the more immediate uterine system. In general, though by no means invariably, the catamenia are suspended and conception is postponed by the process of lactation." (Vol. i, pp. 151-2.)

Dr. Hall must admit that this "extraordinary reciprocal sympathy" is, at least, the most *ordinary* every-day occurrence. It is, in fact, in one sense, only just as "extraordinary" a circumstance as that women conceive and bear children. But this is only one of a hundred examples of the inflated style in which Dr. Hall refers to common physiological facts. It is, however, something extraordinary that Dr. Hall could write the significant words, "the catamenia are suspended and conception postponed by the process of lactation," and yet be, as it would seem, unaware that the facts he mentions demonstratively prove the true sympathy to be between the mammæ and ovaria. After quoting several examples in which this connexion between the two organs had been rendered available in checking uterine hemorrhage, and another in which a maid servant induced the lacteal secretion by putting an infant to her breasts, Dr. Hall infers as follows:

"I would propose, then, that the patient should sleep or one week before and during each catamenial period, with an infant on her bosom." (Vol. i, p. 157.)

Now this remedy *might* possibly be serviceable in certain cases of sterility dependent upon atony of the ovaria; but not because there is sympathy between the mammæ and uterus, but because the irritation of the nipple is usually accompanied with pleasurable feelings, and might, therefore—especially when we consider the means—an infant's lips—act favorably on that portion of the nervous system from which the ovaria derive their tone. But there is not one word said by Dr. Hall as to the *diagnosis* of the cases of aphoria in which it would be useful.

Another remedy need only be stated in the words of Dr. Hall himself; criticism is scarcely needed. As the cold douche relieves inertia of the uterus and so relieves uterine hemorrhage,

"May not a means, obviously of such efficacy against inertia of the uterus, be effectual in those other forms of inertia of the uterine system on which atonic sterility sometimes depends? I would suggest its due administration immediately after intercourse. The actions which lead to conception may be of the excited class. Contraction of the uterus may be excited, and be followed by relaxation and ingurgitation." (Ibid., pp. 157-8.)

And there *may* be people so simple as to try the remedy. A douche of cold water on the hypogastrium "immediately after intercourse!" Dr. Hall is safe here, at least, as to his physiology; the cowleech and stallion-keeper will certainly lay claim to "complete anticipation," as to practice. Here follows the *Io pæan:*

"I trust that it will be admitted that new applications of physiology to practice have been made in this paper; and, first, that the sympathy between the immediate uterine system and the mammæ is both nervous and vascular; and, secondly, that the principles of the reflex actions have been shown to have a practical bearing not previously noticed." (Ibid., pp. 158-9.)

We might multiply examples like the preceding, from the practical papers, as Dr. Hall wishes them to be considered, but we will only say *ex uno disce omnes.* We will therefore turn to his physiological philosophy, and try if we can find anything more satisfactory on his own favorite ground, the nervous system. And here we are sorry to have to make the preliminary remark, that in the numerous papers on this subject in the two volumes, we have almost always to complain of indefinite terms, obscure meaning, and cloudy views. For instance, often as he employs the terms "cerebrum," and "true spinal marrow," he never once defines them in these volumes. A reference (and a troublesome one too, as we know by experience) must be made to previous works for a fundamental proposition which might have been stated at most in two lines.

We have felt embarrassed in selecting a physiological chapter for examination. Many of them are simply repetitions of the ideas already promulgated in the 'Memoirs,' the 'New Memoir,' the 'Lectures,' and the

'Oration,' that Dr. Hall has writ or spoken from time to time; ideas expressed in words full of promise to the ear but frustrating the hope; always going to end in something grand; never soaring from the small circle within which Dr. Hall seems spell-bound. Perhaps some ideas on the nature of sleep will show as briefly as any the easy off-hand style with which Dr. Hall throws out wild suggestions and hints to the rising class of physiologists, and in the same breath adopts the hints as proven verities.

"Little or nothing is yet known of the immediate cause of sleep. I am of opinion, and I shall have to repeat the observation, that a state of contraction of certain muscles of the neck takes place, analogous to that of the orbicularis palpebræ, as sleep comes on; that certain veins are compressed; that congestion of the brain takes place; and, lastly, as a consequence of this last, *sleep*. A similar event takes place for a moment or two, in some cases, inducing that short oblivion, or epilepsy of which Heberden gives so just a picture." (Vol. ii, p. 27.)

It will readily occur to the reader to suppose that Dr. Hall undertakes just to name the muscles that contract and compress the veins, and indicates the veins that are compressed. But he does not. They are "*certain* veins" and "*certain* muscles of the neck." But perhaps he distinctly shows that contraction of muscles actually takes place previously to sleep or a fit of epilepsy? He does not. Then, at least, he proves that there *is* congestion of the brain previously to sleep? No. He stops not for such trifles, but shows that epilepsy, being a disease of congestion, is allied to sleep. He assumes the theory to be proven. Further, as he promised, he repeats the observation:—

"Certain muscles may be muscles principally of excito-motor action; and when volition is withdrawn from the other muscles of the neck, *they* may contract like the orbicularis, and gently compress the jugular veins, and so induce congestion of the brain and sleep—and, as we often observe, attacks of apoplexy and of epilepsy." (Ibid , p. 35.)

But we must not conceal, however, that with regard to this hypothesis, Dr. Hall expressly observes that it "is a mere conjecture, to be accepted for what it is worth, and a mere foil." Then, we say, it is worth just nothing.

But may not a man be allowed to publish a few conjectures? Unquestionably; nevertheless when a prince of philosophers, as Dr. Hall assumes to be, so deeply, too, "imbued with the Baconian philosophy" condescends to conjecture, we have a right to look for something different from this. Volition being withdrawn from certain muscles of the neck, other certain muscles are then allowed to contract and compress veins and cause cerebral congestion, and then sleep comes on. But had not sleep *already* come on when volition was withdrawn from the aforesaid muscles? or, if not, will Dr. Hall gratify his readers by stating from *which* muscles of the neck they must withdraw volition as a preliminary to a good night's rest? An elephant supports the earth, and a tortoise the elephant, but on what does the tortoise stand? Dr. Hall is a veritable Brahmin in his power "fingere hypotheses."

We might multiply examples of this kind; but those we have cited already sufficiently show that Dr. Hall is not able to carry into practice the principles of research he lays down, nor to imitate the "*perfect* model and example" he holds out as a pattern to others.

We are gratified, however, in being able to allow that Dr. Hall has shown, in some of the practical papers, a certain degree of diagnostic and therapeutic acuteness. The statements respecting the uses of enemata of cold and warm water, although not new, are well put. The method described for the regulation of the temperature of a sick room is good, inasmuch as it recommends simple means for the purpose. One important objection, however, occurs to us, viz. that the ventilation of the apartment would be somewhat defective. The precautions necessary to be observed in the treatment of tetanus and hydrophobia have a sound physiological basis, and merit attention; for too much care cannot be given to the prevention of paroxysms, by the exclusion of irritants to the nervous system. We agree with Dr. Hall in thinking that cataplasms are useful in pleurisy—and we have often used them—but not because the atmospheric air is excluded from the skin (as Dr. Hall imagines it to be), but probably because of the action of heat and moisture; there can be, however, no analogy between the treatment of burns and that of pleurisy, with reference to the exclusion of atmospheric air; it is a quaint conceit to think there is. We also think the remarks on the causes and prevention of apoplexy have considerable practical value.

There are many more small practical hints and modes of treatment derived from empirical observation or instructive analogies, which we might go on enumerating. Physicians and practitioners of experience abound in such hints and suggestions, but they do not generally think it necessary to make a book of them, or trumpet them to the world as discoveries. Those of Dr. Hall may be allowed to prove that he is an acute, diligent, and thoughtful physician, but he will be surprised to be told that they do not prove that he is either a Harvey or a Sydenham.

We wish we could, in justice, here hold our pen; but there are two or three things so prominently conspicuous in the manner and style of the volumes before us, that our regard for the dignity of the profession forbids us entirely to overlook them. The first of these which we shall notice, has reference to the singularly dogmatical and boasting tone which the author constantly assumes. As illustrations of this we might refer the reader to numerous passages in the volumes: we shall here only extract the following:

"And here, in reference to my own labours, I trust my readers will allow me to make a very few brief remarks, without imputing to me any other wish than that of doing myself an act of mere justice.

"I may truly affirm that mine has been a really active professional life. In the midst of practice and of lectures, I have allowed few days to elapse without recorded observation. This habit I regard as the *test* of a physician's steadiness and industry. But, besides this, for ten years I devoted a part of my leisure hours—often snatched from hours which should have been devoted to repose—to physiology. My labours in this department have been rewarded by no less a discovery than that of *the Function of the Spinal Marrow, reflex* in its form, *excited* in its character, embracing *all* the *acts* of *ingestion* and of *expulsion* in the animal economy, and therefore all such acts in their relation to the preservation of the individual, and the continuation of the species—a function, therefore, of no mean or inconsiderable extent. To the physiology of the spinal marrow, too, must be added its pathology and therapeutics.......

"But of my various labours, in the course of medical practice, I look upon my researches in regard to the use and abuse of bloodletting, of all remedial measures the most powerful for good and evil, with the greatest satisfaction. I *know* that the Rule for the due administration of bloodletting, which I have adduced from those researches, is the daily means of saving valuable lives; of extricating the individual practitioner from many and most serious difficulties; and of protecting the patient from the dangers of the undue detraction of blood on the one hand, and the inefficient administration of an important remedy on the other.

"I think I may say, of the distinction between the cases of intestinal irritation, of the effects of loss of blood, of inflammation, and of the various *mixed* cases consisting of two or of all three of these, variously mingled together in the same case, pretty nearly what I have said of the Rule for the due and safe administration of bloodletting,—that it is one of great practical importance, and that it has proved the means of saving many dear and valuable lives. This is still more true when the diagnosis in question is confirmed or corrected, and the treatment regulated, by that very rule.

"I remember the time when sporadic puerperal diseases in general were regarded, without due discrimination, as inflammatory, and forthwith treated with lavish bloodletting, by which they were frequently rendered fatal.

"At an early part of my medical career, I published an Essay on this subject, of the practical value of which the late Dr. Baillie spoke in the highest terms. I believe this testimony to have been true. It has constantly been confirmed by those of my friends most engaged in the practice of midwifery, who have had at once the opportunity and the disposition to put my suggestions to the test of experience; and, after a lapse of twenty years, I have still the happiness of thinking that my labours have, in this instance too, had a highly useful result.

"After the rule proposed for the due administration of bloodletting, and the diagnosis established in regard to puerperal diseases, I would rank the observations I have made on that form of hydrocephaloid disease which arises from exhaustion, for their practical utility. I have seen, and do still see, little patients whose maladies are doomed to run a fatal course, under the erroneous idea that they are really hydrocephalus, rescued from their state of peril by a juster diagnosis.

"One other service which I think I have rendered the art of medicine, in its application to infants and children, is that which relates to the treatment of infantile convulsions." (Vol. i, pp. 3-7.)

We do not know what Dr. Hall's ideas may be as to the precise meaning of the words "modest" and "unpretending," by which he characterizes his own lucubrations; all we can say is, that there are many similar passages in the volumes from which we quote; and, in fact, the expressions used by the author, as to the value and merits of his own work, are, in our humble opinion, as far removed from the "modest and unpretending" as can well be conceived.

No doubt Dr. Hall would be shocked to find himself compared, in the remotest possible degree, to the detestable race of puffing charlatans, against whom he is so loudly and so justly indignant in many pages of the volumes before us: and heaven forbid that we should think of making the comparison—even if he had not told us, as he does in his preface, that "a physiologist could not, if he would, either be a quack, or disposed to quackery;"—yet it is painful to observe into what seeming approximation to the objects of his just detestation, morbid vanity and self-conceit can bring an honorable man like Dr. Hall. Like the veriest quack, he addresses to the "general reader," boasts of his industry, his knowledge, his discernment, his discoveries, his great cures. Dr. Hall's enemies —and he admits that he has many—might be disposed to attribute the seeming polarization of meaning in these and similar passages to the desire to produce a reflex action, in a *practical* sense, on "the country practitioner in his lonely drives," or on "the general reader who may be interested in more particular medical subjects;" but we—whom probably, however unjustly, he may reckon among his enemies—are disposed to ascribe all such demonstrations entirely to the egregious conceit and vanity which constitute such a striking feature in Dr. Hall's mental constitution. Yet it must be admitted that it requires the exertion of some candour, and a constant reference to the author's solid merits and high position, to refrain from doubting whether many passages in these volumes were not indited with an aim not strictly scientific. On what may be called the hypothesis of reflex action just hinted at, many things seem readily explicable, which otherwise are puzzling; but with the key of conceit just adverted to in our hands, we find no difficulty with all the seeming allusions or real contradictions. In illustration, let us refer back to our extracts from the paper on Consumption (*supra,* p. 203). Here it might seem that the assertions and cases of *consumption cured* are made suitable to the "general reader;" the doubts as to the efficacy of the remedy and the reality of the cures, to "the lonely driving country practitioner;" but our key unlocks all the difficulties.

Another illustration of the painful peculiarities we are commenting on is to be found in the chapter on scarification of the gums during dentition. He recommends daily scarification of the gums, and even twice daily in urgent cases; "whilst there is fever, or restlessness, or *tendency to* spasm or convulsion." Fever *or* restlessness, or a tendency to!—such is the indefinite language in which Dr. Hall recommends this painful operation to the general reader, student, and "lonely driving country practitioner." The whole is concluded with a grand finale, an Io pæan of mighty success in treating infantile diseases:

"I do not pretend, in the above proposition, to have advanced anything new; but in the *locality* chosen for the operation, and in the *promptitude, repetition, perseverance,* and in the *energy and steadiness of purpose* with which I recommend the measure to be adopted,—if these be fully apprehended,—I believe I do propose something *new;* and when I repeat that since I adopted the plan of *effectually* removing *all* irritation in the gums, stomach, and intestines, in cases of crowing, and other convulsions of the same nature, early enough, I have not known or seen a fatal case, I am aware that I propose a plan of treatment at once new and *invaluable.*" (Vol. i, pp. 33-4.)

The next chapter is an appendix to the preceding, and is on the nature and treatment of stridulous convulsion in infants. The pith of the chapter we found to be the tail-piece. It is a reference to a case published some four or five years ago. "No case could present a more marked diathesis of the convulsive character than this little patient." The results of treatment are stated in letters from the practitioner in attendance, whose name and place of residence are given in full (the two latter particulars we shall venture to omit):

"W—— R——, April 28th, 1842.

"Dear Sir,—I am sure you will be much gratified to hear that your plan of treating the disorder of Mrs. Grey's infant has been signally successful. ........

We have adhered rigidly to the rule prescribed since you saw him. . . . . . . I continued the lancing of the gums twice a day until yesterday, when I only did it once, and the same to-day, as the gums, where divided, continue soft and open, and *slightly ulcerated*, as well as from the amelioration of the symptoms. . . . . . . . I am anxious to have your advice by the bearer how I shall go on, &c.

"W—— R——, May 4th, 1842.

"Dear Sir,—Mrs. Grey's infant has continued to improve since my last report. I believe no spasm has been noticed; the different functions of the body go on in a healthy way. The treatment you suggested has been unremittingly carried out, excepting that, since I last wrote, the gums have been only lanced once a-day." (Vol. i, pp. 44-5.)

We should like to hear what "the lonely driving country practitioner," with his strong common sense, would say to this rubbish; remarkably interesting it is to the lonely driver to learn that, with a healthy nurse, a good appetite, gentle laxatives, change of air, and "sea breezes," the different functions of the body of Mrs. Grey's infant went on in a healthy way; but more interesting we opine it will be to think that this agreeable ordering of the said functions of the body of Mrs. Grey's infant took place *in spite of* the lancing of the gums twice a day, until they were "slightly ulcerated!"

Next comes a chapter on the use of setons; and as usual, when ordered by Dr. Hall, never-failing success attends upon them. *Fiat setaceum,* uttered by him, is the password to health and ease. Example:

"In a variety of cases of acute or chronic, local or limited internal inflammation, I have had recourse to the seton, and uniformly with the most marked success; so that, I think, we may look upon the remedy as almost specific in such cases. It is unnecessary to enumerate them. But hepatitis and nephritis belong to them in an especial manner." (Ibid., p. 48.)

We beg leave to assure Dr. Hall that it *is* necessary to enumerate them; and more necessary still to substantiate his astounding assertion of the seton being used by him "*uniformly* with the most marked success," in local or limited internal inflammation. With the experience we have already acquired (e. g. *in re* the alcoholic lotion) as to the latitude of speech Dr. Hall allows himself to indulge in, we call for evidence.

We pass over one or two unimportant chapters to one headed, "On milk abscess and milk fever." It is, at the least, intended for the glorification of Dr. Hall.

"I am of opinion that what is designated milk-fever is frequently symptomatic of the condition of the mammæ. The remedy for this febrile state also is, therefore, *depletion* of the milk ducts. As a preventive of milk-abscess and milk-fever, and with other hygienic objects, the infant should be put to the mammæ at the moment it is born. If, in spite of this, the mammæ become in the slightest degree tumid, or febrile action be set up, another and a stronger infant should be applied *without delay*. This is *Nature's* mode of relief, and infinitely more efficacious than the application of leeches. The mammæ must be kept absolutely free from heat, tenderness, and tumidity; the patient must take barley-water as her sole nourishment, and the bowels must be freely purged.

"I do not pretend, in the above proposition, to have advanced anything new; but in *proposing such promptitude, perseverance, energy,* and *steadiness,* and *decision of purpose,* with which I recommend the measure to be adopted, if these be fully apprehended, I believe I do propose something *new.*" (Ibid., pp. 74-5.)

It will be observed that this is the identical Io Pæan uttered over the grand gum-lancing aforesaid (see page 197), and may be regarded as the patent formula stereotyped on the great No. X of Dr. Hall's organology. The italics are Dr. Hall's, and of course the qualities he italicizes are rare qualities, especially with the lonely drivers of the country. We imagine one of these—an effeminate creature—wending his way over hill and dale, along lanes and by-roads, suffering from the blast, but gathering strength from 'Observations in Medicine,' and receiving a flash of intellectual light from Dr. Hall's *new* proposition, to keep the bowels of a parturient female open, the mammæ cool, and apply the infant to them as soon as born, or subsequently, with "*promptitude, perseverance, energy, steadiness, and decision of purpose.*"

Another painful feature in the volumes before us, hardly ever seen in the writings of men in the position of Dr. Hall, and one which, like the boasting just animadverted on, savours even more strongly of the spirit of puffery and quackery, is the constant, careful, and, indeed, obtrusive mention of the names and addresses of the general practitioners who have consulted the author, and of the patients for whom he is consulted. We purposely and for obvious reasons omit quoting examples of this undignified proceeding—to give it no worse name; but we can assure our readers that there is hardly ever anything so remarkable or incredible in the cases quoted, or their accompanying circumstances, as to render all this flourish of name, address, and condition in life at all requisite; while in instances in which corroboration or verification would be really valuable, there is a total eclipse of any tangible testimony; as, for example, in the reported cures of phthisis, and of curvature of the spine.

Before closing our remarks on what Dr. Hall is pleased to call his '*Practical* Observations,' we must say a few words on the character of the reprints in the volumes before us. We have stated that one of the avowed objects of Dr. Hall, in bringing out these volumes, is the republication of some of his previous writings. And we may remark that this practice of constantly repeating himself, is a striking illustration as well of the irresistible tendency to self-glorification, so characteristic of Dr. Hall, as of the comparative barrenness or limited range of his intellect. Perhaps there never was an author who persisted so perseveringly and systematically in thrusting the same things — sometimes altered, sometimes unaltered—over and over again on the attention of the public. Every new publication so surely contains a greater or less portion of one or more that preceded it, that we may safely assert, that if all his volumes were simply weeded of repetitions, they would dwindle to less than half their present dimensions. In the volumes before us, this habitual custom of the author is, of course, not foregone. On the contrary, it is carried, as usual, to such excess, that he not only reprints what fairly comes within the compass of his professed subjects, but what has no other relation to these than the relation which is always all-sufficient with Dr. Hall, viz. that the things are his own, or that he thinks they are his own. Even when the subjects of the writings are related, who but Dr. Hall would think of re-reproducing them, well knowing that they have all been already issued and sold to the reader in another form? It is surely somewhat unfair to the purchaser of a book, advertised by the title of 'Practical Observations,' and who bought it solely on the ground of its being what it professed to be, a work on practical medicine, to have forced on him chapter after chapter of old physiological memoirs and lectures, essays on hybernation, reports of policemen at coroner's inquests (vol. ii, p. 301), extracts from letters (ib., p. 308), reports of Dr. Hall's speeches at societies (p. 298), &c. &c. And it is not over-civil to take our money anew even for practical papers which we had already paid for, sometimes more than once, in Dr. Hall's previous publications, or in publications to which he contributed, without giving us previous warning of what we had to expect in our new purchase. For, instance, in the last of the two volumes now before us, we have nearly one third of the matter—actually six or seven of the largest chapters in it—transcribed, almost verbatim, from the 'Cyclopædia of Practical Medicine' (to which Dr. Hall contributed the article on puerperal diseases), only enlarged by the addition of a few cases—probably omitted by the editors in the printing of the original papers.

And here we are compelled still further to animadvert on the extremely careless manner in which many of these reprints have been made. So precious in Dr. Hall's eyes is everything that Dr. Hall fancies he can call his own, that he seems almost to lack the heart to destroy any part of what he has once fathered, though demonstrably bad or wrong. Accordingly, we find, in these volumes not a few of the old writings reproduced in their original form, although this may be .one which harmonizes very badly with the new forms with which the writings are now surrounded. In fact, it often happens that the old and new matter directly contradict each other; or that the old matter is so literally old, as to have lost all relationship to the existing state of knowledge.

In illustration of what is just said, we need only turn to the chapters on puerperal diseases already referred to. These essays, we do not hesitate to denounce as being utterly behind our present state of knowledge of the subject. For example, at pp. 120, 128-29, vol. ii, Dr. Hall gives us three seats of puerperal inflammation in the abdomen, but seems innocently ignorant of its being most frequent in the uterine veins and lymphatics. It would actually seem that *uterine phlebitis* was unknown to him! At pp. 122 and 127 he speaks of pelvic abscess as "partial inflammation and suppuration of the *peritoneum.*" Now, the truth is, as every one but Dr. Hall knows, that the seat of these abscesses is the cellular substance behind the peritoneum, and not the peritoneum itself; the disease is no more partial peritonitis than a malignant whitlow is inflammation of the skin of the finger. Various other instances of the same sort of antiquarian lore might be extracted from the same chapters; and we could lay our finger on as many direct contradictions in the old and new papers. Thus, at pp. 131-32, vol. ii, the "utility and necessity" of purgatives in peritonitis is laid down as "undoubted;" that is old. At p. 9, however, it is declared that this same treatment in this same disease is prescribed (says the author) "in vain, but not, I believe, with safety and impunity;" this is new.

As we have already hinted, a large portion of the volume has no reference whatever to *practical* medicine. For instance, the chapters on the "Inverse ratio of irritability," &c., and on "Hybernation." The first of these, which occupies twenty-three pages, from page 223 to 245, is printed verbatim from the 'Philosophical Transactions.' Yet, even this reprint is so slovenly executed, that at pages 228, 229, 230, there are references by letters, &c., to a diagram which does not exist in the volume, but for which the author acknowledges in a note he is *compelled* to refer the reader to the 'Philosophical Transactions.' The whole of this essay, excepting the first five and the last two paragraphs, Dr. Hall had already once before reprinted, verbatim from the original, in his article on "Irritability," in Dr. Todd's 'Cyclopædia of Anatomy'—where, however, as well as in the 'Philosophical Transactions,' the anxious reader may find the diagram.

The chapter on "Hybernation," which occupies forty-one pages of this new series of *Practical Observations in Medicine* (from page 248 to page 289), is in like manner a verbatim reprint, with all the errors of the original. We have taken the trouble to examine this embalmed relic, not only with the original memoir in the 'Transactions of the Royal Society,' but also with its *double* in the 'Cyclopædia of Anatomy.' The article in the Cyclopædia consists of only twenty-three columns and a half, or a little more than eleven pages, yet *three fourths* of the whole are a verbatim reprint of Dr. Hall's Memoir in the 'Philosophical Transactions!' Among the new matter contained in this article, we have a formal correction of one of the chief views of the original memoir, viz. that "*nervous sensibility* is not impaired during hybernation." This he states in the Cyclopædia to be an error, explaining that, what he originally attributed to sensibility is of the "*excito-motory kind.*" And yet our readers will hardly believe, that in this second series of the present so-called *Practical Observations in Medicine*, Dr. Hall has reprinted in 1846 every word of his memoir of 1832, extending over forty-one pages, and has added a postscript of eleven lines, to show that views which occupy two pages of this very reprint are erroneous, although he had corrected formerly in the Cyclopædia the words now reprinted, yet repudiated!

In regard to these reprints, it will further strike the reader who knows the facts of the case, as curious, that, in by far the greater number of instances, no indication is given in the body of the work, nor in the table of contents, that they are reprints at all,—all of them being formally introduced under a consecutive numeration of Chapters, as if they had appeared for the first time, and without any reference (with one or two exceptions) to the work or works in which they had previously appeared! Whether this singular incommunicativeness of the author be the consequence of design or of mere carelessness, we will leave for others to say: we content ourselves with denouncing the whole as one of the most remarkable instances of what is vulgarly called *Bookmaking* that we have ever met with.

We would fain here close our notice of these volumes, and with it all further remarks, for the present, on the literary and scientific merits and claims of Dr. Hall; but the eternal trumpeting of his own great deeds in the matter of physiology, particularly the physiology of the nervous system, and specially in regard to the reflex function, and his reiterated charges against ourselves, compel us to add something on this subject.

In every work Dr. Hall has published, in almost every scrap he has printed, and, of course, in the volumes before us (see *supra*, p. 211), he has over and over again, and loudly, proclaimed his own unequalled merits in this department, and scoffed at the pretensions of other excellent men, his predecessors or contemporaries. Knowing Dr. Hall's undoubted merits, grateful for what he has really done in this branch of physiology and therapeutics, we have been willing to overlook much that we knew to be unjust and wrong, both in his pretensions and imputations. But the same sense of justice to others and to ourselves, which has led us in the preceding pages to illustrate and expose some of his therapeutical vagaries and vanities, has at length induced us, however reluctantly, to break our silence once more on the subject of his physiological discoveries. On the present occasion we shall confine ourselves to the endeavour to rescue from unmerited obloquy and disregard one illustrious name, which Dr. Hall has so pertinaciously and ungenerously sought to deprive of its due honours. Our available space, our time and inclination, forbid our now doing more than this, though there are other names also, both of the living and the dead, which may justly demand a like vindication.

On former occasions we took some pains to notice the physiological views of George Prochaska. In our Fifth Volume we reprinted the whole of his chapter on the functions of the Common Sensory; and in our Ninth Volume we discussed, more at length, the claims to priority of neurological discoveries, in an historical retrospect of the whole subject. We then pointed out the real meaning of the terms used by Whytt and by Prochaska, and maintained that on a due consideration of the true value of those used by Prochaska, his doctrines did, in fact, constitute, as we stated, "a complete anticipation" of Dr. Hall's doctrine of a reflex function. "It is perfectly evident," we said, "that Prochaska had a very distinct idea of the function of the spinal cord and medulla oblongata as a centre of reflected action; and that he specified the principal classes of these, referring them all to the excitement of impressions. We do not see," we added, "what essential difference there is between this doctrine and that of the *Reflex Function* of the spinal cord, as *first* propounded by Dr. M. Hall." (p. 115.) Since that period Dr. Hall—when he has condescended to mention them—has taken every opportunity to depreciate the merits of Whytt and Prochaska, as if he were impelled, by the conviction that his struggles for fame would be valueless, unless he scornfully trampled their claims under foot. We may add, that Dr. Hall has never forgiven us our tribute to the fame of the professor of Prague, and has never ceased from attempts to force from us a concession which truth has hitherto forbidden and still forbids us to make. He is always about to say to us his final farewell, but casts long lingering looks upon us, as if his very immortality depended upon the opinions expressed in this journal. In his work on 'Diseases and Derangements,' he parts with us, he "trusts, for ever," with the declaration that we have "sacrificed truth, honour, and justice on the altar of pert folly, vanity, and insolence;" and yet in his quarto volume he reprints nearly the whole of the chapter quoted by us from Prochaska. Not satisfied with this, he again reverts, in the second series of his present 'Observations' to "ill-natured and disreputable inuendoes about Whytt, Prochaska," &c. &c.

and finally pours forth the bitterness of his soul against us in a letter published lately in one of the weekly journals.*

Every reader of this Review is aware that the opinions expressed in it are fortified, as much as is possible, by trustworthy facts and statements; and doubtless it is from a knowledge of this that Dr. Hall estimates our opinions so highly; but every reader is equally aware that they are, after all, only opinions which he may at his good pleasure either admit or reject, and for which, if they be erroneous or unjust, we are responsible to the tribunal of public opinion. It is a grievous charge that Dr. Hall brings against us before this tribunal; it is a charge to which we boldly plead not guilty; and as truth, honour, and justice are as dear to us as to him, we hope the reader will bear with us if we occupy his attention for a brief space, in vindicating the fair fame of a deceased philosopher; and in vindicating him make our own defence.

And first we will let Dr. Hall state his own objects and discoveries, and we shall allow him to do so in their last perfected form, although it is obvious that we could have made our case much stronger by confining ourselves, as we might justly have done, to his earliest promulgated views:—

"My real objects have been—

"5. First, to separate the reflex actions from any movements resulting from sensation and volition.

"6. Secondly, to trace these actions to an acknowledged source or principle of action in the animal economy—the *vis nervosa* of Haller acting according to *newly-discovered laws.*

"7. Thirdly, to *limit* these actions to *the true spinal marrow,* with its appropriate *incident and reflex nerves,* exclusively of the cerebral and ganglionic systems.

"8. Fourthly, to apply the principle of action involved in these facts to *physiology,* viz. to the physiology of *all the acts of exclusion, of ingestion, of retention,* and *of expulsion* in the animal frame.

"9. Fifthly, to trace this principle of action in its relation to pathology, viz. to the pathology of the *entire class of spasmodic diseases;* and,

"10. Sixthly, to show its relation to *therapeutics,* and especially to *the action of certain remedial, and certain deleterious physical agents.*

"11. Finally, it is to these objects, taken as a *whole* or as a *system,* that I prefer my claims: and I do not pretend that an occasional remark may not have been incidentally made by some previous writer, bearing upon some one or more of them." (New Memoir on the Nervous System, p. 5. Lond. 1843.)

How much of these views belong to Prochaska in the opinion of Dr. Hall is stated very specifically by that gentleman in the work just quoted:

"19. Having on a former occasion (Memoir II, p. 53.) sufficiently refuted the

* We subjoin a part of this effusion: it illustrates well the singular constitution of Dr. Hall's mind; "Dr. Forbes, as editor of the *British and Foreign Medical Review,* inserted a paragraph with the following heading,—'*Complete Anticipation of Dr. Marshall Hall by Prochaska.*' I know not in what allowable terms I can express my indignation, after the lapse of so many years, at this most malignant calumny. In the first place, it is totally unfounded in truth; in the second, Dr. Forbes might have known this by a comparison of a few intellectual moments of Prochaska's work, and my own; in the third, such a comparison was due to his own character (I should think) as well as to my own; fourthly, he must have received the paragraph from some ignorant and very malignant person, and should, therefore, have taken double pains to make that comparison. Lastly, Dr. Forbes has submitted, during a long series of years, to be viewed by a brother in the profession (nay, by many) as a false calumniator, without coming forward with either justification (a thing impossible), or confession (a thing which should have been spontaneous and immediate), when the calumny was properly exposed," &c. (Lancet, Aug. 15, 1846, p. 200.)

claims of several writers, both of remote and recent date, in regard to the subject of my labours, I shall only, in this place, make a very few and brief remarks on the imagined similarity between the views of Prochaska and my own.

"20. Let any one compare the distinct detail of my views just given in sections 5-11, with the observations of Prochaska, and he will at once discover that there is *nothing* in that author possessing *the most remote similarity* to those views." (Ibid. p. 7.)

Dr. Hall then gives long extracts from Prochaska's volume in proof of his statement "that there is *nothing* in that author possessing the *most remote similarity*" to the views quoted above, and numbered five to eleven consecutively. The italics in the extract are Dr. Hall's, and we trust that our readers will remember this emphatic declaration when they have perused the subjoined translation of the fourth chapter, third fasciculus, of the 'Adnotationes Academicæ,' written and published before Dr. Marshall Hall was born.*

On the occasion already referred to (vol. IX, p. 114) we remarked that the term *sensorium commune* would not be understood if reference were not made to the meaning attached to certain words when Prochaska wrote. "In modern times," we then observed, "the term *sensorium commune* is applied to the part of the nervous system where *impressions* become *sensations;* and if we set out with this notion, we should find it difficult to interpret any part of his (Prochaska's) doctrines; but he takes care to define his meaning in the very first paragraph, where he explains the *sensorium commune* to be that part of the nervous centres at which *external impressions* conducted to it by different nerves, give rise to certain and determinate *motions* by respondent motor nerves." We added, "To avoid confusion, we might substitute the term *centre of reflexion* for sensorium commune;" better, perhaps, if we had said "we might substitute the term, *true spinal marrow,* according to the nomenclature of Dr. Hall," the sole difference being, as we shall see presently, that Prochaska's Sensorium Commune included a little more than Dr. Hall's completed "true spinal system:" we say *completed* system, because we believe Dr. Hall's field of reflex action, as first laid out by him (in 1832) comprehended quite as large an extent as Prochaska's. Thus, in his first paper communicated to the Zoological Society, he tells us, "The peculiarity of this motion consists in its being excited by irritation of the extreme portion of the sentient nerves, whence the impression is conveyed through the corresponding portion of *brain and spinal marrow, as a centre,* to the extremities of the motor nerves;" and he there speaks of what he afterwards (1836) termed the excito-motor power as "a property which attaches itself to any part of an animal, the corresponding portion of *the brain and spinal marrow* of which is entire." (Proceedings of the Zoological Society, Nov. 27, 1832.) The subsequent greater limitation of the field of reflexion by Dr. Hall was so far a decided improvement on the doctrine he previously held; but this had nothing to do with what alone concerns us at present, the great fundamental principle of the doctrines—the reflex action or function of the spinal marrow. We are, therefore, fully authorized to request the reader, in perusing the translation, to regard as synonymous not only these two terms, but also the other terms which hold the same relative place in the two versions of the doc-

* Georgii Prochaska, m.d., et Professoris Anatomiæ, &c., in Cæsareo-Regia Universitate Pragensi, Adnotationum Academicarum Fasciculus Tertius. Pragæ, 1784.

trine, the Prochaskean and Hallean. Thus, he must read *common sensory* as *true spinal marrow; sensorial* nerves as *afferent* or *incident-excitor; motor* nerves as *reflex-motor*, &c. If he do so, and if it should happen that he reads this chapter for the first time, we think he will not be surprised that we have felt it to be a paramount duty, if we would preserve the character of impartial critics, to endeavour to set things in their proper light.

To avoid all risk of unfairness in the comparison we are about to institute between Dr. Hall's views and Prochaska's, we shall, in the first place, give a simple translation of the extracts from the Professor's works. We at first thought of introducing, within parentheses, the synonymous modern terms; but we leave this for the reader to do for himself. We will only add, that if our translation reads stiffly, it is because, in making it, we have been desirous that no imputation should be thrown upon our accuracy, which might perhaps have been the case if we had rendered the author's meaning in freer language.

"Chapter IV. The functions of the common sensory.

"§ 1. *What is the common sensory, what its functions, and what its seat?*

"The external impressions which are made on the sensorial nerves are very quickly propagated throughout the whole length of the nerves, as far as their origin: when they [the impressions] have arrived there, they are reflected by a certain law, and pass on to certain and corresponding motor nerves, through which, being again very quickly propagated to muscles, they excite certain and definite motions. This locality, in which, as in a centre, the sensorial nerves, as well as the motor nerves, meet and communicate, and in which the impressions made on the sensorial nerves are reflected on the motor nerves, is designated by a term, now adopted by most physiologists—the Common Sensory.

"The most distinguished men have not fixed on one place as the seat of the common sensory. Bontekoe, Lancisi, De la Peyronie have placed the common sensory in the corpus callosum; Willis derived the perception of sensation and the source of movements from the corpora striata; Des Cartes attributed the function of the common sensory to the pineal gland; Vieussens to the centrum ovale; Boerhaave decided that aggregate of points to be the common sensory, in which all the sensory nerves terminate, and from which all the motor nerves arise, and accordingly placed it in the medulla fornicata, surrounding the cavity of the ventricles. In a later work, *de Morbis Nervorum,* Boerhaave places the common sensory in the boundary line of the medullary and cortical substance, which opinion Tissot thought to be extremely probable, regarding it as confirmed by the observations of Wepfer. Mayer seems to place the common sensory in the medulla oblongata; that distinguished man, J. D. Metzger, appears to be also of this opinion; the celebrated Camper said, that if the common sensory has a seat at all, it ought to be in the pineal gland, and in the nates and testes, and that, therefore, the opinion of Des Cartes was not so very absurd. It certainly does not appear that the whole of the cerebrum and cerebellum enters into the constitution of the common sensory, which portions of the nervous system appear rather to be the instruments that the soul directly uses for performing its own actions, termed animal; but the common sensory, properly so called, seems not improbably to extend through the medulla oblongata, the crura of the cerebrum and cerebellum, also part of the thalami optici, and the whole of the medulla spinalis, in a word, it is coextensive with the origin of the nerves. That the common sensory* extends to the medulla spinalis is manifest from the motions exhibited by decapitated animals, which cannot take place without the consensus and intervention of the nerves arising from the medulla spinalis; for

* Marherr contends that the medulla spinalis ought also to be referred to the common sensory, in Prælect. ad Instit. Med. Boerhaavii, tom. ii, p. 404.

the decapitated frog, if pricked, not only withdraws the punctured part, but also creeps and leaps, which cannot be done without the consensus of the sensorial and motor nerves, the seat of which consensus must necessarily be in the medulla spinalis—the portion remaining of the common sensory.

"The reflexion of sensorial into motor impressions, which takes place in the common sensory, is not performed according to mere physical laws, where the angle of reflexion is equal to the angle of incidence, and where the re-action is equal to the action; but that reflexion follows according to certain laws writ, as it were, by Nature on the medullary pulp of the sensory, which laws we are able to know from their effects only, and in no wise to find out by our reason. The general law, however, by which the common sensory reflects sensorial into motor impressions, is the preservation of the individual; so that certain motor impressions follow certain external impressions calculated to injure our body, and give rise to movements having this object, namely, that the annoying cause be averted and removed from our body: and, *vice versa,* internal or motor impressions follow external or sensorial impressions beneficial to us, giving rise to motions tending to this end, namely, that the agreeable condition shall be further conserved. Very many instances which might be adduced undoubtedly prove this general law of the reflexions of the common sensory, of which it may be sufficient to adduce a few. Irritation being made on the internal membrane of the nostrils excites sneezing, because the impression made on the olfactory nerves by the irritation, is conducted along them to the common sensory, there by a definite law is reflected upon motor nerves going to muscles employed in respiration, and through these produces a strong expiration through the nostrils, whereby, the air passing with force, the cause of the irritation is removed and ejected. In like manner it happens that when irritation is caused in the trachea by the descent of a particle of food or a drop of fluid, the irritation caused is conducted to the common sensory, and there reflected on the nerves devoted to the movement of respiration, so that a violent cough is excited,—a most suitable means for expelling the cause of irritation—which does not cease until the cause of irritation be ejected. If a friend brings his finger near to our eye, although we may be persuaded that no injury is about to be done to us, nevertheless the impression carried along the optic nerve to the common sensory is so reflected upon the nerves devoted to the motion of the eyelids, that the eyelids are involuntarily closed, and prevent the offensive contact of the finger with the eye. These and innumerable other examples which might be brought forward, manifestly show how much the reflexion of sensorial impressions into motorial, effected through the common sensory, has reference to the maintaining of the conservation of our body. Wherefore, Tissot enumerates the action of the common sensory amongst those powers, the sum and co-ordination of which constitute the nature of our living body.

"Since, therefore, the principal function of the common sensory consists in the reflection of sensorial impressions into motor, it is to be noted, that this reflexion may take place, either with consciousness or without consciousness. The movements of the heart, stomach, and intestinal canal depend in no way upon consciousness; yet, forasmuch as no muscular movement can be excited, unless a stimulus applied to the sensorial nerves passes by a peculiar reflexion to the motor nerves, and excites contraction of the muscle, it is certain that the reflexion of the impressions suitable for exciting those movements, if it takes place in the common sensory, is effected without consciousness. But it is a question whether these impressions, in order that they may be reflected, do really travel so far as the common sensory, or are, without taking this long circuit, reflected nearer, in the ganglia, from whence these parts derive many nerves? This matter is further to be considered afterwards. But that reflexions of sensorial impressions into motor are effected in the common sensory itself, the mind being utterly unaware, is shown by certain acts remaining in apoplectics deprived entirely of consciousness: for they have a strong pulse, breathe strongly, and also raise the hand, and very often unconsciously apply it to the affected part. The common sensory also acts independently of consciousness in producing the convulsive movements of epileptics, and also those which are sometimes observed in persons buried in profound sleep, namely, the retractions of pricked or irritated limbs,—to say nothing of the motion of the heart and the respiratory acts. To this category also belong all those motions which remain some time in the body of a decapitated man, or other animal, and are excited when the trunk, and particularly the medulla spinalis are irritated, which motions certainly take place without consciousness, and are regulated by the remaining portion of the common sensory existing in the medulla spinalis. All these actions flow from the organism, and by physical laws peculiar to the common sensory, and are, therefore, spontaneous and automatic. The actions taking place in the animal body, with attendant consciousness, are either such as the soul has no voluntary control over, or such as the soul can restrain and prohibit at pleasure; the former being governed by the common sensory alone, independently of the soul, are as much automatic as those of which the soul is unconscious. Of this character are sneezing from an irritant applied to the nostrils, cough from an irritant fallen into the trachea, vomiting from a titillation of the fauces, or after taking an emetic; the tremors and convulsions in St. Vitus's dance, and in a paroxysm of intermittent fever, &c. Those actions, however, which the soul directs or limits by its own power, even although the common sensory has its share in producing them, are nevertheless called animal and not automatic; concerning which we treat in the next chapter." (pp. 114-20.)

The views here detailed are too definite and too lucidly expounded to be considered for one moment as nothing better than random hints or guesses; and, accordingly, we find them repeated by the author, in the text-book of his class at Vienna,* published in 1802; and again in another physiological work, published in 1820, and repeated with the same, or even greater lucidity, after a lapse of thirty-six years:

"That part of the nervous system is termed the general sensory [allgemeine Sensorium] in which external impressions meet, and from which internal impressions are distributed to all parts of our body, where, consequently, the accord [Uebereinstimmung] of all organs necessary to life, takes place, and where external impressions are reflected, according to the law of self-preservation, with or without consciousness, into inner impressions, or those operating outwardly [nach aussen]. The sensory, in which the impressions with consciousness are reflected, may be termed the mental or soul-sensory [Seelensensorium], and the other the corporeal sensory [körperliche Sensorium]. Willis termed the one the reasoning and the other the corporeal soul. The seat of the soul-sensory is particularly [vorzüglich] the brain; of the body-sensory, the spinal marrow; and also, as it appears, the nervous plexuses and ganglia. The last is shown to be the case by acephalous monstrosities, which sometimes for many hours, and even a whole day, remain alive, move their limbs, utter cries, suck the nipple, and the like. So also decapitated animals are observed to continue for a few moments to perform movements with design. By virtue of this accord of the nerves, the operation of a stimulus is not limited to the nerves immediately irritated, but is extended to remote nerves and their organs, which is named the *consensus nervorum*; as, for example, the irritation of the gravid uterus excites nausea, vomiting, headache, toothache, and the like." (Physiologie, oder Lehre von der Natur des Menschen, von Dr. u. Prof. Georg Prochaska, &c. &c. 8vo, Wien, 1820, p. 92)

Let us now briefly consider the views here stated, and compare them with those of Dr. Hall. In the first place, we find that Prochaska *did* "separate the reflex actions fro many movements resulting from sensation

* Lehrsätze aus der Physiologie des Menschen. Von D. Georg Prochaska, ordentl. Lehrer der Anatomie, Physiologie und Angenarzneykunde in Wien, &c. &c. Zum Gebrauche seiner Vorlesungen. Zweyte verbesserte und vermehrte Auflage. Wien, 1802.

and volition." He makes this distinction repeatedly, and in terms so positive and so clear, that there can be no mistake about the matter. This separation by Prochaska is the first of the views respecting which Dr. Hall most emphatically asseverates, "there is *nothing* in that author possessing the *most remote similarity !*"

We find, too, that Prochaska *did* limit these reflex actions to a part of the nervous system, namely, that part comprising the medulla spinalis, the medulla oblongata, the crura of the cerebrum and cerebellum, and part of the thalami optici; Dr. Hall's "true spinal marrow" comprises, even in its last development, the corpora quadrigemina, the medulla oblongata, and the medulla spinalis. Prochaska also discriminated between the reflex, the intellectual, and the ganglionic centres. As regards the reflex and intellectual, the quotation already given is decisive; the subjoined contains his views respecting the ganglionic; it is from the section headed, "*An nervi in gangliis suis consentiunt ?*"

"Unzer* and Winterl taught that external impressions are reflected in the ganglia as they are reflected in the common sensory, and that the ganglia are special [particularia] sensories, which opinion appears to be by no means destitute of probability. For, considering that the minute and invisible nerves disseminated on the internal membrane of the heart and of its auricles, receive a stimulus from the inflowing venous blood,—if they cannot transmit the impression of that stimulus to the common sensory through the ganglia of the intercostal nerve [the sympathetic],† and yet communicate it to the motor nerves distributed to the fleshy structure of the heart and auricles,—there is necessarily a consensus of the sensory nerves on the inner membrane of the heart with the motor nerves distributed through the fleshy structure of the heart and auricles, which (consensus) must have its seat either in the ganglia of the intercostal [or sympathetic] nerve, or below them, in the conjunctions and communications of the nerves. It seems, therefore, probable that besides the common sensory which we conjecture to be in the medulla oblongata, medulla spinalis, pons varolii, and crura of the cerebrum and cerebellum, &c., there exist special sensories in the ganglia and plexuses (concatenationes) of the nerves, in which (special sensories) external impressions ascending along the nerves are reflected, without needing to ascend to the common sensory, in order to be there reflected." (p. 29.)

Prochaska also applied the principle of action of his "common sensory" to physiology, beautifully and clearly pointing out the general law by which it regulated its operations, namely, the law of preservation of the individual; not simply by the avoidance of the painful, but by attaining the pleasurable. He also applied his views to the illustration of pathological phenomena, as those of chorea Sti Viti, &c., mentioning especially the tremors or rigors, and convulsions which occur during the paroxysms of intermittent fevers; and this, we think, a most important application of the doctrine of reflexion overlooked altogether by Dr. Hall.

With these plain statements before them, we leave our readers to draw their own conclusion on the question, as to whether Dr. Hall's assertion be correct, or whether it presents even the smallest approach to accuracy.

* The works of Unzer, viz. his 'Grundriss eines Lehrgebäudes von der Sinnlichkeit der thierischen Körper,' published in 1768, and another, published in 1771, entitled 'Erste Gründe einer Physiologie der eigentlichen thierischen Natur thierischer Körper,' are very remarkable productions, of which we hope to give some account on a future occasion. Although the doctrines contained in them have an intimate relation with some parts of the present discussion, we purposely avoid referring to them, as we wish to confine ourselves, for the present, strictly to the defence of Prochaska.

† The sympathetic nerve was formerly termed the great intercostal nerve.

Is it TRUE that there is *nothing* in the writings of Prochaska possessing the *most remote similarity* to his own views, for such we have seen is the unqualified, emphatic assertion of Dr. Hall? We will add one or two other random assertions, just as audacious, and just as untenable. The first is made immediately after extracts from the chapter just translated:

"It is surely needless to add another remark upon this author. I will therefore only further observe, or rather repeat, that we have not, in *any* author, the idea of the true *reflex* action of the *vis nervosa*, or of a reflex physiological act; that no one had detected the *reflex* nature of the physiological acts of *respiration*, or discovered the system of *incident* respiratory nerves," &c. (New Memoir, p. 10.)

We shall revert to the assertion respecting these claims to the discovery of "the idea of the true *reflex* action of the *vis nervosa*" and of the respiratory muscles; all we need ask is this: Is it not staringly apparent that there *is* an idea of a *reflex physiological act* in Prochaska's illustrations, derived from the acts of sneezing or coughing? Does not Dr. Hall himself reckon them among the acts of "*expulsion?*"

We add yet another assertion as companion to the above:

"It would be easy to select passages from Whytt and Prochaska to whom so much reference has been made of late, to show their profound ignorance of the nervous system, as it is now understood. It is not necessary for me to add any commentary. Both these authors have, doubtless, great merit; but they were both entirely and absolutely unacquainted with the distinct and exclusive *physiology* of the true spinal marrow, in the acts of ingestion and of egestion, in their relation to the preservation of the individual, and the propagation of the species." (Observations, 2d Series, p. 16.)

And this monstrous assertion is writ in the face of Prochaska's clear and lucid announcement, "THE GENERAL LAW by which the Common Sensory reflects sensorial impressions into motor is *the preservation of the individual*—EST NOSTRI CONSERVATIO!'*

We subjoin yet another example of Dr. Hall's reckless disregard of historical truth:

"My First Memoir was entitled 'On the reflex *function* of the medulla oblongata and medulla spinalis.' This important *function*, as the nervous agent in all the Acts of Ingestion and of Egestion in the animal economy, was previously unknown. It is *not* mentioned by Whytt, or Prochaska, or any other author; who, however, they may cite the term reflex, or detail experiments, or treat of sympathetic actions, have not, I affirm, associated *one physiological act* with any such reflex *function* of the spinal marrow. This is, therefore, my discovery." (New Memoir on the Nervous System, p. 9.)

If the emphatic term in this sentence had been spinal marrow, we could have supposed that Dr. Hall meant to play upon that term, and that as Prochaska used the term common sensory, Dr. Hall's statement might be verbally although not essentially correct. But the emphatic term is "reflex *function*," and if Prochaska has not associated the physiological acts of vomiting, sneezing, coughing (from an impression on the

* He even mentions this physiological law of preservation twice, in as many words, in the same chapter, as will be seen in our translation. We here subjoin the original: "Hæc et innumera quæ afferri possent exempla manifeste ostendunt quantopere *reflexio sensoriarum impressionum in motorias* per sensorium commune facienda *conservationem nostri corporis* respiciat." (p. 118.) "Generalis tamen lex, qua *commune Sensorium impressiones sensorias in motorias reflectit*, EST NOSTRI CONSERVATIO." (p. 117.) We may add also, that in the physiological works subsequently published by him, he continually repeats this general law, and as one of fundamental importance.

sensory nerves) with a "reflex *function*" appropriated to a certain portion of the central axis, then words have no meaning, and language conveys no ideas.

We might stay our pen here, for we feel convinced that the preceding proofs of *our* truthfulness are unanswerable, and must bear conviction to the minds of the most prejudiced reader. And as we remarked respecting the "practical observations," so we have to remark respecting Dr. Hall's pretensions to physiological discovery; *ex uno disce omnes.* We need not, therefore, trouble our readers with further exposures of Dr. Hall's conduct to other physiologists, his predecessors and contemporaries, because it is abundantly manifest that no reliance whatever ought to be placed on his assertions when his vanity is concerned, and how seldom is it not! We may, however, revert to one other assumed discovery of Dr. Hall's, because *that* belongs also, as well as the "reflex function," to George Prochaska.

Dr. Hall states in his new memoir "that we have not in *any* author the idea of the true reflex action of the vis nervosa," and further adds:

"I have been always struck by three remarkable facts. The first is, that there should have been a principle of muscular action in the animal economy, acknowledged amongst physiologists, the *vis nervosa* of Haller, without any application to physiology. The second, that there should have been a series of experimental phenomena—certain muscular actions—untraced, either *backwards* to any *principle* of action, or *forwards* to any *physiological* act. The third, that there should have been a series of functions, and a class of diseases hitherto unexplained, which the excito-motor principle, just noticed, and involved in those experiments, should be calculated to explain in the most perfect and satisfactory manner." (New Memoir, p. 6.)

Now Dr. Hall terms this *vis nervosa* the excito-motor power, and arranges the portions of the nervous system according as they are endowed or unendowed with this vis nervosa; or in other words, are excitor or in-excitor. We will quote Dr. Hall, to prevent mistake:

"The nervous system may be divided into different portions, according as they are endowed or unendowed with the excito-motor power; thus, in general terms, the cerebrum and cerebellum are *in*-excitor; the medulla oblongata and medulla spinalis are excitor; the nerves of special sense are *in*-excitor; the trifacial and the analogous special nerves are incident excitor nerves; the nerves distributed to muscles are direct excitors; the ganglionic system is excito-motor, but, for reasons which will be given hereafter, in a less prompt and energetic degree." (New Memoir, p. 17.)

The discovery of the vis nervosa is not claimed by Dr. Hall, nor of the fact that it acts from the central ganglia to the muscles; the fact was already too well known. The discovery he claims is of its application to physiology, and of its action from the surface along a special class of nerves to the central ganglia, and there being reflected on the motor nerves. Let us now then examine Prochaska's views in relation to this point. The title of the eighth section of chapter I of the Fasciculus from which we have already quoted,—and the title alone indicates what Dr. Hall denies to any other writer, viz., an extended application of the vis nervosa to physiology—thus runs:

"*The functions of the nervous system are explained by the vis nervosa.*

"At length also in this department of animal physics we abandon the

Cartesian method of philosophizing, and embrace the Newtonian, being persuaded that the way to truth is tedious, and truly most doubtful through hypotheses and conjectures, but far more certain, excellent, and short that which is analytical—(*quæ a posteriori ad causam ducit*). Newton has given the name of *attraction* [vis attractiva] to the inscrutable cause of physical attractions, observed its effects, discovered and arranged its laws of action, and so has established a doctrine useful and honorable to the human mind; thus also ought we to act in reference to the functions of the nervous system. We will term the cause inherent in the pulp of the nerves there producing its effects, and hitherto not determined, the *vis nervosa;* its effects which are the functions of the nervous system, having observed, we will arrange—we will discover their laws; and by this method we may be able to found a true and useful doctrine, which will certainly afford a new light and a more elegant form to medical art. Haller has already used the term *vis nervosa* for the purpose of indicating the agent by which the nerves effect contractions of muscles; but J. A. Unzer has thrown the greatest light on this matter; for although he still may use the term *animal spirits* to make himself more readily comprehended, his system is quite independent of any belief in the existence of these, as he himself explains." (pp. 28-9.)

Here it is manifest that Prochaska has in view a much wider application of the vis nervosa than that adopted by Haller; he applies it to the whole nervous system, and not to the motor portion only, and exults, with the prevision of a seer, over the results which will follow the application of his views to medicine. "They will surely," he declares, "afford new light and give a finer form to medical art." This we esteem a singularly touching trait in Prochaska's mind, and of itself is a token of deep and powerful thought, and of a clear conviction of the truth of his doctrines. His candour towards Unzer, too, marks a generous nature; would that in our day it had met with a more generous requital!

The seat of the vis nervosa was fixed by Prochaska in the medullary pulp of the nerves and cerebro-spinal axis; it was latent there, and required to be excited by a stimulus. Just as the iron requires to be struck by a flint to elicit a spark, so, he says, the vis nervosa is latent and produces no actions of the nervous system until a stimulus be applied. The stimulus may be either material or immaterial, mechanical or mental. (p. 47.) He then proceeds to treat of the vis nervosa in subsections. Two of these, the 4th and 5th (pp. 49, 56) are headed "*Under what conditions the vis nervosa is increased and diminished*"—titles, by the way, which naturally remind us of an expression used by Dr. Hall in his first paper in the Philosophical Transactions for 1833, viz. "*that the reflex function admits of exaltation and diminution,*" (p. 651); though, of course, Dr. Hall will find no similarity between the two. The author enumerates several pathological states in which the increase takes place, &c. He thus explains convulsive attacks:

"If the vis nervosa be increased in the common sensory, there results, firstly, that the external impressions made on the sensory nerves and carried to the common sensory, are too suddenly and violently reflected and pass on to the motor nerves, and thus excite movement and convulsions contrary to the will of the soul, as happens in the frights of infants, and also of some adults who are terrified by some trifling crash or noise." (p. 52.)

He also considers the circumstances under which it is diminished. (§ 5, p. 56.) He further states (§ 6, p. 60) that it is divisible, for it exists in nerves when separated from the central axis by ligature or section, and in the spinal cord when separated from the brain; quoting, in illustration, experiments on decapitated frogs, the muscular contractions of the heart when separated from the body, and the vital acts of acephalous fœtuses. Prochaska expressly states that when a nerve is divided it retains the power of exciting muscular contractions when irritated, but cannot excite sensations; but he emphatically observes, that nothing departs from the nerve by section, and it only loses the power of exciting sensation because the power of propagating impressions to the brain is taken away by the severance of continuity. (p. 68.) His views as to the vis nervosa of the nerves are very clear; it must, however, be remembered that the nerve spoken of by him is a *compound* nerve.

"If an impression be made on the extremity of a nerve, termed an external impression, it is most quickly propagated along the whole length of the nerve as far as its origin; and, *vice versa*, if an impression be made on the origin of the nerve, termed an internal impression, this also most quickly descends along the nerve to its termination; but if the impression be made on the trunk of a nerve it is at the same moment quickly propagated both to the origin and termination. This fitness of the nerves to receive impressions and quickly transmit them, either way along their whole length, ought to be called the *vis nervosa* of the nerves; it is also properly called the *sensibility* or *mobility* of nerves, or, as Unzer also rightly termed it, the *corporeal sense without concomitant perception*." (p. 76.)*

Prochaska expressly declines defining the nature of this vis nervosa as to whether it be electricity, or phlogiston, or some kind of air, or the material of light; he leaves that question to others to decide. Nor will he say positively whether this *vis* is derived from the numerous blood-vessels accompanying the nerves, or is drawn through inorganic pores from the air, &c.; but he insists that it is *not derived from the brain*, but is a something conjoined with the medullary pulp, which the nerves, however they acquire it, acquire in the same way as the brain itself. (p. 77.) He is positive, too, as to "the equal function of the nerve in exciting sensation and motion; namely, to receive the impression of a stimulus, and to pass it quickly along its whole length; which impression, when it comes to the brain, causes a perception of a sensation, but if to the muscle excites its contraction." (p. 78.)

The following extract shows that Prochaska considered this vis nervosa the agent in reflecting impressions. It opens the fifth chapter devoted to the cerebral functions, and we may here observe that Prochaska not only clearly proves the brain to be the organ of the mind, and distinct from the common sensory or "true spinal marrow," but he devotes a section to the special consideration of the question, whether it be a compound organ, and decides in the affirmative: such was his "profound ignorance of the nervous system as now understood"!

"In that part of the nervous system which we have termed the common sensory, there inheres such a mechanism that the external sensorial impressions of

* It is obvious from the context, that the word *sensibility* (sensibilitas), as here used by Prochaska, meant, not the capacity of sensation or consciousness of impressions, but merely an inherent power of respondence to impression. The word is continually used in this sense by the French and German Physiologists at the present time; and Prochaska's acceptation of the term *sensorium commune* is in harmony with this.

the nerves are reflected in it upon the motor nerves, in a singular manner and by peculiar laws, so that they excite certain and determinate muscular acts. It has been previously stated that many truly automatic motions in man are excited solely by the vis nervosa of the common sensory; and many animals live and are regulated by this vis nervosa of the common sensory alone, which animals are destitute of brain or the higher animal endowments, and may therefore be truly termed *automata ;* but to the nervous system of man, and many animals having an affinity with him, is superadded the brain, and besides this a certain principle which we term the mind or soul, an Ens of incorporeal origin," &c. (p. 130.)

After this, can we regard the vis nervosa of the common sensory of Prochaska, as different in any essential respect from the excito-motor power of Dr. Hall? To be sure the latter considers it the *sole* property of the true spinal system; but whether this be an improvement on Prochaska's theory or not remains to be seen. It is clear, however, that, according to Prochaska's views, the vis nervosa gives the nerves and spinal marrow the property of receiving and transmitting impressions, which, when conducted to the brain, cause sensation, and when transferred from afferent to muscular nerves, or directly applied to muscular nerves, excite motion; and that, therefore, they are endowed with an excitor power; and that the vis nervosa is the excito-motor power.

How clearly and precisely Prochaska distinguished between the functions of the brain and true-spinal system, and between the acts of the vis nervosa excited by the soul or the will, and the acts of the vis nervosa or excito-motor power of the common sensory excited by impressions, will be demonstrated by another extract, and this, we hope, will be considered sufficient. In the last quotation, it would be observed that Prochaska termed the reflex acts *automatic,* because they were dependent upon the mechanism of the common sensory or "true spinal marrow" acted on by its vis nervosa or "excito-motor power."

"§ IV. *What movements are properly termed animal* [*voluntary*] *?*

"The muscular acts of the human body are of two kinds according to their exciting cause; thus, one class is called animal or voluntary, because the soul willing and commanding, they may be excited, increased, diminished, or checked; the other is involuntary of which the soul is unconscious, or if conscious, they are performed against its will and are excited by a mere mechanical corporeal stimulus applied to the nervous system ; which movement is, therefore, termed spontaneous and automatic. Nerves are required for the production of both kinds of motion. But nerves do not act without a stimulus, which [stimulus] is either afforded by the soul, willing, or—it being unconscious or unwilling,—by any substance applied to the nerves. From whence it is evident that those movements only should be termed animal, which depend upon the free command of the soul; which it excites or checks by its own free will ; on the contrary, those movements which in no wise depend on the volition of the soul, but are performed when it is either unconscious or unwilling, cannot be called animal, but are purely mechanical and automatic.

"Observation teaches that there are muscles in the human body over which the soul has no control whatever, and which are moved automatically during the whole life : of this kind are the heart [ventricles], auricles, œsophagus, stomach, intestinal canal, and among these also the movements of the iris may be reckoned. Other muscles there are, which ordinarily during life respond to volitions, and are, therefore, termed voluntary : such are the muscles of the limbs, trunk, head, face, eyes, tongue, genitals, with which also the sphincters of the anus and urinary bladder may be included. Sometimes, however, it happens that all these muscles refuse submission to the soul, and, without its concurrence or consciousness, are powerfully and preternaturally agitated by some mechanical stimulus, which thing may be observed to occur in the convulsions of hysterical females, of epileptics, of infants, of those suffering from St. Vitus's dance; and these movements, although effected by voluntary muscles, cannot be otherwise regarded than as automatic. These muscles are not as yet moved voluntarily, but for the most part automatically in the fœtus in utero or the newly born; the cerebrum at this early age is not sufficiently developed, nor until the organs of the faculty of thought are successively evolved, does the soul attain the power to think and to use the muscles subject to its volition. To automatic movement belongs also the lifting and application of the hand to the head by apoplectics, the turning of the body in sleep, and in some degree somnambulism itself, but which, however, it appears, should also be partly ascribed to obscure sensations and volitions, of which the soul becomes immediately oblivious. Thirdly, there are muscles which perpetually act independently of the volitions of the soul, excited solely by a mechanical stimulus, over which muscles the soul has, however, a free voluntary power, being able *pro libitu*, to accelerate or retard their motions, or even to check them for a time. The action of these muscles we call a mixed action. Such are the muscles subservient to respiration, which almost constantly act automatically, but over which there is a free power of the soul, so that it is able to accelerate or retard respiration, or even to stop it for a while. But if the mechanical stimulus be too powerful, then, although the soul be unwilling, the muscles of respiration are excited to action; as for example, when a crumb gets into the trachea the most violent cough is excited, which cannot be restrained by any effort of the soul; so also the soul cannot restrain sneezing when the pituitary membrane of the nostrils is excited by an acrid stimulus." (pp. 144-46.)

Dr. Hall may say that this is a jumble of incoherent facts, and especially that the movements of the heart, stomach, and intestines belong to the ganglionic system. But this opinion Dr. Hall himself seems to have adopted very lately, abandoning his previous division into cerebral, true spinal, and ganglionic; while Prochaska himself (as we have already seen) doubted whether the sympathetic ganglia might not be special centres of reflexion, quite independent of the great central axis of reflexion; yet Dr. Hall makes no mention of Prochaska in that change of opinion.

"The intra-spinal structure is also the central organ of the ganglionic system· I formerly supposed that the ganglia were themselves the centre of this system—an opinion founded on an experiment, the accuracy of which I am now led to doubt." (Observations in Medicine, 2d Series, p. 57.)

And on the strength of that solitary experiment, Dr. Hall not only deduced a general principle, but ridiculed the doctrines of a man in every way his superior,—whose doctrines he copies, and whose identical doubts he shares!

Such is the imperfect exposition we must, for the present, content ourselves with making of the despised doctrines of George Prochaska. But imperfect as it is, it is sufficient to justify us in stating the general result of our inquiry to be a much more complete anticipation than we have hitherto declared, and that all the *fundamental* and *acknowledged* views which are claimed by Dr. Hall as exclusively his own, are to be found in Prochaska's writings most succinctly, and most clearly set forth. There is first, the vis nervosa or excito-motor principle demonstrated, and its laws of action laid down; its excitation by acts of will in the cerebrum ; its excitation by impressions made on afferent nerves leading to the common sensory; its excitation by reflection of impressions on motor or efferent nerves; its presence in the nerves; and its share in their functions. The cerebral system is distinguished as the seat of will and of intellect, from another central axis, the seat or centre of reflexion, and the limits of the two systems are accurately defined. The distinction is broadly made between voluntary and reflex acts, and motions which may be produced in either way; the exciting causes of the two classes are lucidly pointed out, and it is shown that the will is the excitor of the action of the vis nervosa in voluntary acts, while a mechanical or material stimulus is shown to originate the reflex acts. The distinction between reflex acts with, and reflex acts without consciousness, is well and most plainly drawn; and the influence of the will in restraining or modifying the reflex movements is clearly indicated. Thus respiration is shown to be an excited act, and, therefore, ordinarily and habitually, reflex in its nature, but that the respiratory muscles are under some control of the will, and, therefore, it may be modified by volition when required. Lastly, the general law or final cause of reflex action—the preservation of the individual, *nostri conservatio*—is most distinctly stated and most admirably illustrated.

Do we, then, mean to accuse Dr. Hall of having been acquainted with Prochaska's writings before the publication of his neurological views, and, consequently, of knowingly promulgating another man's doctrines as his own? We do not. Dr. Hall has distinctly denied all such knowledge; and we have no proof that he had it. Wherefore, we can only say, that, however discreditable it might be to Dr. Hall, as a scholar, to have been unacquainted with so striking a work as that of the Professor of Prague, published to the world so long before his time, and on the very subject of his own inquiries, we must still charitably believe that he was ignorant of its existence at the time he first promulgated his views relative to the Reflex Function, although he was acquainted with the writings of Whytt, Blane, &c., containing a good deal of analogous matter. We consequently acquit Dr. Hall of all serious blame in this respect. We merely say that he was culpably ignorant of what he ought to have known, and that he had the misfortune—not the fault—to be entirely anticipated, in all the essential part of his doctrines, by others, and especially by Prochaska. So stood the case, we may admit, in 1832, or even in 1833, the date of Dr. Hall's first Memoir. But how stands it now? How has it stood since 1835, the period at which Dr. Hall acknowledges he became acquainted with the writings of Prochaska?. How, more especially, has it stood since April 1838, when we, in this Journal, and, about the same time, Mr. George, in the 'Medical Gazette,'* first disclosed to physiologists the claims of the Professor of Prague, and forced them more particularly on the attention of Dr. Hall? Very different indeed: to Dr. Hall disgracefully different: inasmuch as, instead of acknowledging the merits of his predecessor, when made known to him, or admitting, in any degree, the

* Med. Gaz., April 7th and 14th, 1838. In these clever papers the author has given a very striking and, in some respects, even a more complete exposition of the identity of the views of Prochaska and Dr. Hall, than we have given in the present article. Mr. George's essay is well worthy the attention of all who are interested in the present discussion, or in the history of neurology.

similarity of his doctrines to his own—a similarity, to use no stronger term, which it is impossible for any unprejudiced person to deny—he has either studiously passed over in silence or openly ridiculed and maligned Prochaska's doctrines, and poured on the heads of those who did no more than assert their resemblance to his own, all the venom which his bitter nature could engender. And up to the very hour at which we write, he continues, as we have seen, to boast, as loudly as ever, of his originality; although for more than ten years he has, by his own showing, been familiar with the very writings which we have proved to contain not merely the elements but the developed germs of his early doctrines, and from which he has demonstrably drawn not a few of the materials wherewith he has since so greatly modified them. We can add no comment in words that can in any way emulate, in damning potency, the eloquence of this simple statement. It is grievous to be forced to write it down; it is melancholy to contemplate its full import: we shall therefore turn from the painful theme, and hasten to conclude our task.

After all this, it will naturally be asked, what is due to Dr. Hall? What can he justly claim? What are his deserts? We reply, that much is due to him; and how much, we, of all men, are best entitled to say, seeing that we have devoted more time, and space, and labour in unfolding his undoubted claims in this Journal, than have been accorded to him by all the other Journals of Europe put together. It is no subject of concern to us, that Dr. Hall has somewhat ungraciously received our humble but well-meant aid, because it was mixed with homage to others. We only did what we believed to be our duty, although we are well aware that some of the best physiologists of our time have considered our deference to him as too profound, and our commendations as too highly coloured. We do not share in this judgment; we still think as we have heretofore thought; and the frequency with which we have dwelt on this theme, is our sole reason for not reiterating our opinions here. In sober earnestness, indeed, we think, it is impossible to contemplate Dr. Hall's actual position in the estimation and regard of his contemporaries and fellow-labourers in science, without a compassionate sympathy which is at once melancholy and distressing. Instead of being, as, perhaps, he might have been, the head and leader of the physiologists of his country, he lives the very Pariah of the physiological caste, the Ishmael of a desert created and sought out by himself, with his hand against every man and every man's hand against him. How much better would it have been for his fame—how much better, we should think, for his happiness—had Dr. Hall had the fortune or the wisdom to have chosen another course, and studied his own heart; and, if not gifted by Nature with a genial or generous temperament, had at least learned, as he might have learned, to discipline his rebellious propensities in the school of a better philosophy. Then would he have found his contemporaries willing and happy to yield him more than he claimed; would have seen that, by allowing merit to others, he increased his own; and would have known nought of that worst bitterness to a vain-glorious man, of being slighted by those on whose breath he lives, and compelled, in expiation of unjust pretensions, to look to the tribunal of posterity for the recognition even of the merits which he knows to be his own.

